# Revisiting the hemispheric asymmetry in midlatitude ozone changes following the Mount Pinatubo eruption: A 3‐D model study

**DOI:** 10.1002/2015GL063052

**Published:** 2015-04-21

**Authors:** S. S. Dhomse, M. P. Chipperfield, W. Feng, R. Hossaini, G. W. Mann, M. L. Santee

**Affiliations:** ^1^ School of Earth and EnvironmentUniversity of LeedsLeedsUK; ^2^ National Centre for Earth ObservationUniversity of LeedsLeedsUK; ^3^ National Centre for Atmospheric ScienceUniversity of LeedsLeedsUK; ^4^ Jet Propulsion LaboratoryCalifornia Institute of TechnologyPasadenaCaliforniaUSA

**Keywords:** Pinatubo, ozone, chemical modeling, satellite data

## Abstract

Following the eruption of Mount Pinatubo, satellite and in situ measurements showed a large enhancement in stratospheric aerosol in both hemispheres, but significant midlatitude column O_3_ depletion was observed only in the north. We use a three‐dimensional chemical transport model to determine the mechanisms behind this hemispheric asymmetry. The model, forced by European Centre for Medium‐Range Weather Forecasts ERA‐Interim reanalyses and updated aerosol surface area density, successfully simulates observed large column NO_2_ decreases and the different extents of ozone depletion in the two hemispheres. The chemical ozone loss is similar in the Northern (NH) and Southern Hemispheres (SH), but the contrasting role of dynamics increases the depletion in the NH and decreases it in the SH. The relevant SH dynamics are not captured as well by earlier ERA‐40 reanalyses. Overall, the smaller SH column O_3_ depletion can be attributed to dynamical variability and smaller SH background lower stratosphere O_3_ concentrations.

## Key Points

- Model successfully simulates NO_2_ and O_3_ changes after Pinatubo eruption
- VSLS chemistry amplifies ozone losses by about 2%
- ERA‐Interim data helps to improve representation of NO_2_ and O_3_ losses

## 1 Introduction

The eruption of Mount Pinatubo during June 1991 in the Philippines (15°N) injected between 14 and 20 Tg SO_2_ [*Guo et al.*, 2004] into the stratosphere which was largely converted into H_2_SO_4_. Subsequently, a significant increase in stratospheric aerosol was observed by satellite and in situ measurements in both hemispheres [*Stratospheric Processes and their Role in Climate (SPARC)*, 2006]. Such volcanically enhanced stratospheric aerosol can affect the climate system in three ways. First, an increase in shortwave backscattering by small aerosols can lead to a decrease in tropospheric temperatures [*McCormick et al.*, 1995]. Second, accumulation and coagulation lead to larger aerosol particles, which warm the stratosphere by absorption of long‐wave radiation, thus enhancing the tropics‐to‐pole temperature gradient, increasing tropical upwelling, and modifying local circulations [*Young et al.*, 1994]. Third, an increase in heterogeneous chemical processing perturbs stratospheric NO*_y_* and activates Cl*_y_* species, leading to chemical O_3_ loss [*Fahey et al.*, 1993].

Using Total Ozone Mapping Spectrometer (TOMS) data, *Gleason et al.* [1993] reported a 2–3% decrease in global O_3_ (60°S–60°N) after the eruption, primarily in the Northern Hemisphere (NH). Also using TOMS data, *Randel et al.* [1995] reported a column O_3_ decrease of up to 4% at low latitudes during 1992. They also estimated large (6–10%) O_3_ depletion in the NH but negligible depletion in the Southern Hemisphere (SH). The reason(s) for these negligible post‐Pinatubo O_3_ losses in the SH has remained a scientific question for the last two decades [*World Meteorological Organization (WMO)*, 2011]. This is because satellite measurements indicated similar aerosol enhancements in both hemispheres [*SPARC*, 2006], and ground‐based observations also showed large NO_2_ decreases, indicative of aerosol processing, in both hemispheres [e.g., *Koike et al.*, 1994; *Van Roozendael et al.*, 1997].

Many chemical modeling studies have failed to simulate the observed interhemispheric asymmetry in the O_3_ depletion. *Solomon et al.* [1996] used a 2‐D chemical‐dynamical model but their simulated O_3_ losses were 50% smaller than the observations. They argued that this discrepancy might be due to the neglect of very short‐lived species (VSLS) chemistry, or weaker transport of O_3_‐depleted air from the polar vortex to midlatitudes. By including an extra 8 parts per trillion (ppt) of VSLS bromine in their 2‐D model, *Salawitch et al.* [2005] could simulate the magnitude of O_3_ losses in the NH midlatitudes, but their model overestimated O_3_ losses in the SH midlatitudes. *Fleming et al.* [2007] also used a 2‐D model, nudged with National Centers for Environmental Prediction/National Center for Atmospheric Research (NCEP/NCAR) and European Centre for Medium‐Range Weather Forecasts (ECMWF) ERA‐40 reanalysis data. In the SH midlatitudes their 2‐D model showed good agreement with the observations, but in the NH, modeled O_3_ losses were almost 50% smaller than observations. These studies show that simulating the observed O_3_ changes in both hemispheres using chemical processes alone is not possible.

Using the SLIMCAT 3‐D chemical transport model (CTM), forced with UK Met Office analyses and climatological stratospheric aerosol surface area densities (SAD), *Hadjinicolaou et al.* [1997] and *Chipperfield* [1999] could simulate column O_3_ losses in the NH midlatitudes. Hence, they argued that dynamical changes played an important role in enhancing O_3_ losses in the NH compared to the SH. *Feng et al.* [2007] also used SLIMCAT (forced by ERA‐40 analyses) and found that inclusion of VSLS chemistry led to an overestimation of O_3_ losses over the post‐Pinatubo period in both hemispheres. Using GEOS‐Chem, a 3D‐CTM forced with Goddard Earth Observing System (GEOS) data, *Stolarski et al.* [2006] could simulate smaller O_3_ losses in the SH, but GEOS‐Chem could not simulate large NH O_3_ losses. *Telford et al.* [2009] used the United Kingdom Chemistry Aerosol (UKCA) Chemistry‐Climate Model (CCM), nudged with ERA‐40 data and found that the model simulated nearly identical midlatitude chemical O_3_ depletion (~10 Dobson units, DU) in both hemispheres and about 20 DU and 10 dynamical O_3_ depletion in the NH and SH, respectively.

Recently, 18 free‐running CCMs participated in the CCMVal‐2 activity, but none of them could simulate the observed interhemispheric asymmetry in O_3_ depletion [*SPARC*, 2010, chapter 8]. Again, this points to chemical forcing not being the sole cause of the O_3_ changes and their hemispheric asymmetry. Meanwhile, *Poberaj et al.* [2011] analyzed NCEP/NCAR reanalysis data and found anomalous (large) wave activity in winter 1991 and 1992. They argued that smaller O_3_ losses in the SH midlatitudes are mostly due to a stronger Brewer‐Dobson (BD) circulation along with aerosol‐induced local heating in the stratosphere that caused an increase in O_3_ transport from the tropics to the middle to high latitudes [e.g., *Dhomse et al.*, 2006] in 1991 and 1992. Using GEOS‐CCM simulations, *Aquila et al.* [2013] also suggested that aerosol‐induced heating and subsequent changes in the BD circulation must have counteracted chemical O_3_ loss in the SH.

Using the Canadian Middle Atmosphere Model 3‐D CCM, nudged with ECMWF reanalysis data (ERA‐40 and ERA‐Interim), *Shepherd et al.* [2014] showed reasonable agreement between modeled and ground‐based total O_3_ observations in both hemispheres. Their model did capture the hemispheric asymmetry in the post‐Pinatubo midlatitude O_3_ loss, although it slightly underestimated the magnitude. However, their CCM did not perform well in simulating high‐latitude SH O_3_ loss, which can affect midlatitudes by export of vortex air, and it also ignored VSLS species.

Overall, the results of the previous studies summarized above are somewhat inconclusive. They point to the importance of both chemical and dynamical changes. The magnitude of midlatitude O_3_ changes will also depend on an accurate simulation of polar O_3_ loss and is affected by the inclusion of the known abundance of VSLS bromine. No study has so far successfully treated all of these processes.

Here we use the updated TOMCAT/SLIMCAT 3‐D CTM forced with ECMWF reanalysis data. The main aim of this study is to provide an analysis of the stratospheric O_3_ changes in the presence of Pinatubo‐enhanced aerosol using a state‐of‐the‐art CTM with the latest meteorological data. We also attempt to identify possible failings in previous modeling studies based on earlier versions of meteorological reanalyses. Therefore, we performed model simulations with two different reanalysis data sets (ERA‐40 and ERA‐Interim), two different versions of SAD and with/without VSLS chemistry. For comparison we use TOMS/SBUV (Solar Backscatter Ultraviolet Instrument) merged total O_3_ data, as well as NO_2_ total column data from two midlatitude stations (Lauder, 45°S and Jungfraujoch, 46°N). Simulated O_3_ profiles are compared against Microwave Limb Sounder (MLS) and Halogen Occultation Experiment (HALOE) satellite data.

## 2 Model Setup

We use the TOMCAT/SLIMCAT CTM with different aerosol and meteorological forcings. A detailed description of the model can be found in *Chipperfield* [1999, 2006] with latest updates related to this study in *Dhomse et al.* [2011, 2013]. A key improvement since *Dhomse et al.* [2013] is that the photolysis scheme now uses model‐calculated O_3_ profiles at each grid box rather than climatological profiles as used previously. We have used a model resolution of 5.6° × 5.6° with 32 *σ‐p* levels from the surface to ~60 km. The chemistry scheme includes a detailed description of the O*_x_*, NO*_y_*, Cl*_y_*, Br*_y_*, and HO*_x_* families, as well as source gases. The model also includes the brominated VSLS CH_2_Br_2_ and CHBr_3_, which yield an additional 6 pptv of stratospheric bromine [*Hossaini et al.,* 2013]. The model includes a treatment of heterogeneous reactions on sulfate aerosols and polar stratospheric clouds.

In this study, we present results from nine simulations with different aerosol, chemical and dynamical conditions (also see the supporting information). In run A_v1SAD the model was forced with 6‐hourly ERA‐Interim reanalysis data and SAD data from *SPARC* [2006] (hereafter v1) for the 1979–2000 time period. Runs B_v2SAD and C_climSAD were similar to A_v1SAD but used SAD from *Arfeuille et al.* [2013] (hereafter v2) or climatological monthly mean SAD values (from 1996 to 2005), respectively. To compare our results with previous studies, run D_era40 was identical to run A_v1SAD but forced by ERA‐40 reanalyses. To diagnose the VSLS contribution to O_3_ loss in the presence of volcanic aerosol, runs E_novsls1 and F_novsls2 were identical to runs A_v1SAD and B_v2SAD, respectively, but without VSLS bromine. Runs G_91dyn, H_92dyn, and I_93dyn were similar to A_v1SAD but used annually repeating 6‐hourly meteorological fields (ERA‐Interim) for years 1991, 1992, and 1993, respectively.

## 3 Data

For total O_3_, we use TOMS/SBUV merged data obtained from http://acd‐ext.gsfc.nasa.gov/Data_services/merged/. These are constructed by merging individual TOMS, SBUV, and SBUV/2 total O_3_ data. We also use total column O_3_ data from ground‐based instruments archived by the World Ozone and Ultraviolet Radiation Data Centre (WOUDC). Here we use zonal mean monthly mean data constructed using filter, Dobson, and Brewer Spectrometers (ftp://ftp.tor.ec.gc.ca/Projects‐Campaigns/ZonalMeans/gb_1964‐2010_za.txt). NO_2_ total column data for Jungfraujoch and Lauder were obtained from the Network for the Detection of Atmospheric Composition Change (NDACC) website ftp://ftp.cpc.ncep.noaa.gov/ndacc/station/. O_3_ profile data from HALOE (v19) and MLS (v5) instruments on Upper Atmosphere Research Satellite were obtained via http://mirador.gsfc.nasa.gov/.

## 4 Results and Discussion

**Figure 1:**
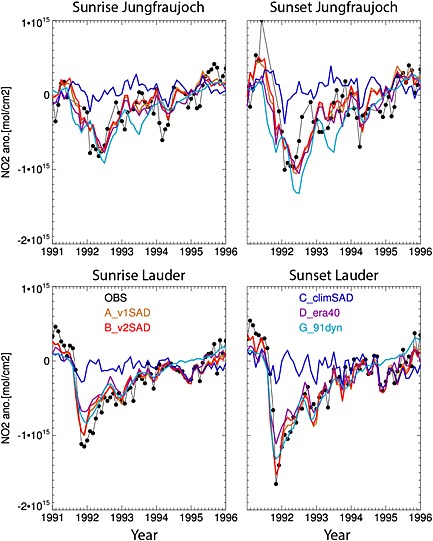
Monthly mean anomalies in total column NO_2_ (molecules/cm^2^) from (left) sunrise and (right) sunset measurements at Jungfraujoch (46°N, top) and Lauder (45°S, bottom). The NO_2_ column anomalies from model runs A_v1SAD (orange), B_v2SAD (red), C_climSAD (blue), D_era40 (violet), and G_91dyn (light blue) are also shown. Anomalies are calculated by subtracting the 10 year (1990–1999) monthly means.

NO_2_ is a key member of the stratospheric odd‐nitrogen family (NO*_y_*), and its abundance depends on several gas phase and heterogeneous reactions. It has a large diurnal cycle through conversion to N_2_O_5_, which can then be converted to HNO_3_ on aerosols. This leads to decreases in stratospheric NO_2_ under conditions of high aerosol loading. Figure 1 compares observed and modeled sunrise and sunset column NO_2_ anomalies at Lauder and Jungfraujoch. Overall, there is good agreement between the model runs with enhanced volcanic aerosols and observations. At Lauder runs A_v1SAD and B_v2SAD capture the decreases in NO_2_ of up to 40% in October 1991 compared to October 1990 [*Johnston et al.*, 1992] and steady recovery afterward. At Jungfraujoch, the modeled NO_2_ columns again capture the largest (up to 40%) NO_2_ decreases during NH spring 1992, but the agreement is not as good as at Lauder. This poorer agreement could be due to the wintertime NH BD circulation, which is dynamically more active and shows larger annual and interannual variabilities than in the SH.

*Dhomse et al.* [2014] noted that v2SAD is about 10‐50% smaller than v1SAD in both hemispheres between 1991 and 1993. However, modeled NO_2_ anomalies from A_v1SAD are very similar to those from B_v2SAD at both stations, which is likely due to the “saturation effect” discussed in *Fahey et al.* [1993]. At Lauder, the modeled NO_2_ columns from runs A_v1SAD and B_v2SAD show up to 10% less NO_2_ decrease compared to the observations, which is within the measurement uncertainties. Again, even with large differences between v1 SAD and v2 SAD in 1991–1992 [*Dhomse et al.*, 2014], NO_2_ differences between these two simulations at Jungfraujoch are not significant.

The NO_2_ anomalies from runs C_climSAD and G_91dyn represent variability due to dynamical and chemical processes, respectively. At Lauder anomalies from both runs A_v1SAD and B_v2SAD converge toward C_climSAD in 1994, suggesting that aerosol‐induced perturbations in the SH stratospheric NO_2_ lasted for about 3 years. In contrast, at Jungfraujoch NO_2_ anomalies from A_v1SAD and B_v2SAD approach values from runs C_climSAD only in 1995, suggesting an even longer perturbation (~4 years) in the NH.

**Figure 2:**
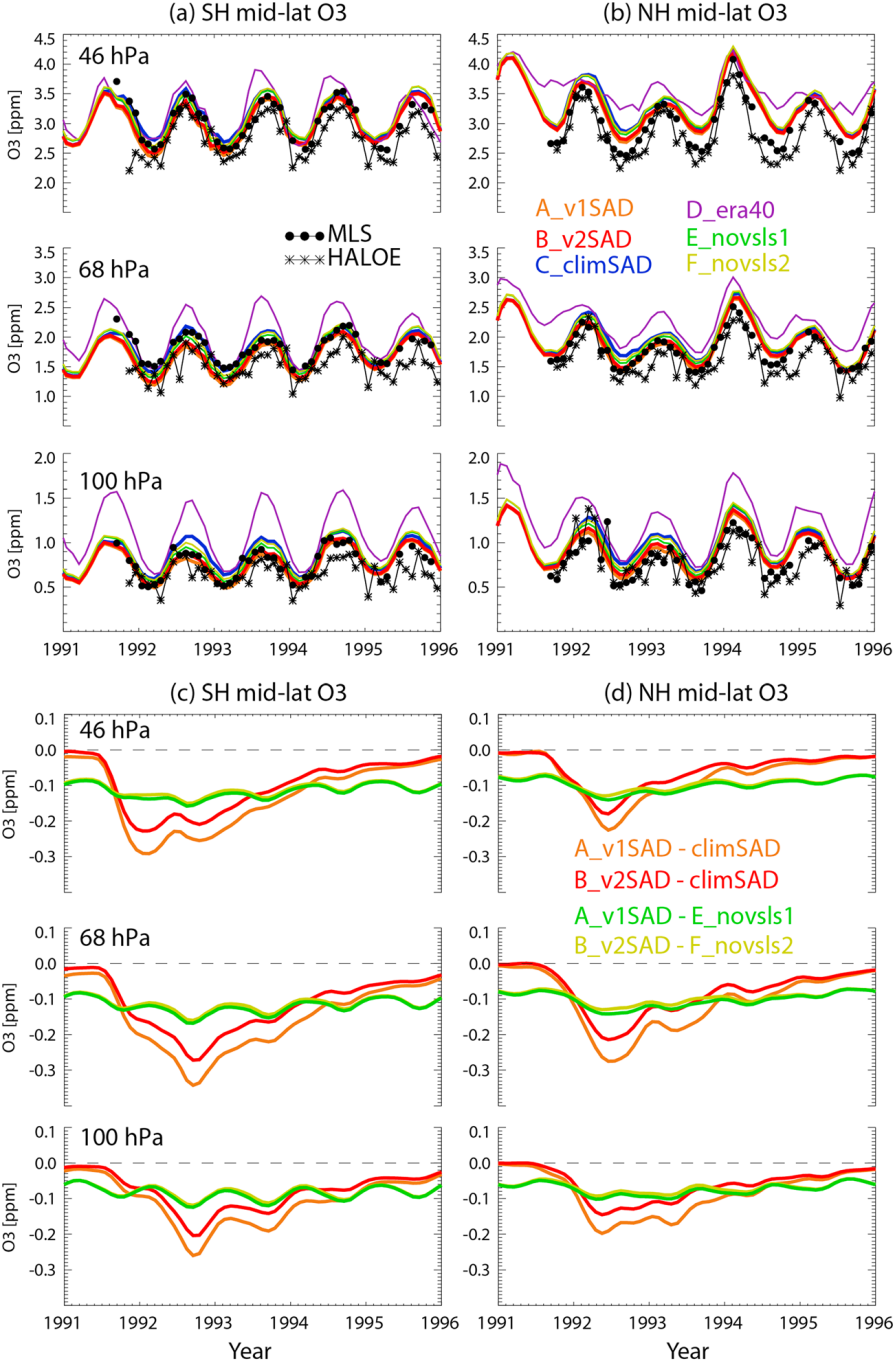
Time series of monthly mean O_3_ volume mixing ratio (ppm) at 46, 68 and 100 hPa for (a) SH (35°S–60°S) and (b) NH (35°N–60°N) midlatitudes. Monthly mean O_3_ from MLS (filled circles) and HALOE (stars) are shown with black symbols. The model runs are shown by the colored lines. (c, d) Corresponding O_3_ differences between selected model simulations are shown.

Figure 2 shows a time series of NH and SH midlatitude (35°–60°) monthly mean O_3_ mixing ratios at 100, 68 and 46 hPa from six model simulations and satellite data. The simulations using ERA‐Interim clearly perform much better than run D_era40. Run D_era40 also has an annual cycle which is much too large and an overall positive bias in O_3_ which is up to 50% in the lower stratosphere and largest in winter/spring. This is another example of the known stratospheric transport errors in ERA‐40 data [*Monge‐Sanz et al.*, 2007]. Overall, there is reasonable agreement between the ERA‐Interim simulations and observations, especially in the SH. The agreement is worse in the NH, and at 46 hPa, in particular, the model overestimates the observations. There are differences between the MLS and HALOE observations, possibly related to the much sparser coverage of HALOE, and generally, the model agrees better with MLS.

At 100 hPa the ERA‐Interim runs and observations indicate that in the NH, the amplitude of the annual cycle and the annual mean O_3_ values are about 0.2 ppm (up to 15%) larger than those in the SH. These smaller background O_3_ concentrations in the SH lower stratosphere are consistent with the weaker strength of the BD circulation during austral winter than boreal winter.

Following the Mount Pinatubo eruption (15°N) during June 1991, differences between runs C_climSAD and A_v1SAD (or B_v2SAD) start to become distinct in September in the SH and November in the NH of that year. These differences, which quantify the chemical O_3_ loss, are slightly larger in the SH, and the timing of the maximum loss is different. For example, at 100 hPa, runs A_v1SAD and B_v2SAD show the largest chemical O_3_ losses (0.25 and 0.20 ppm, respectively) in September 1992, whereas the maximum NH O_3_ losses (0.20 and 0.15 ppm) occur in March 1992. As expected, O_3_ mixing ratios from A_v1SAD are slightly smaller than those from B_v2SAD. In general, run B_v2SAD seems to show relatively better agreement with MLS measurements in the SH, but A_v1SAD agrees better in the NH. Therefore, our simulations suggest that the v1 SAD might be positively biased in the SH and therefore that previous studies using this SAD data set might have overestimated SH O_3_ losses.

**Figure 3:**
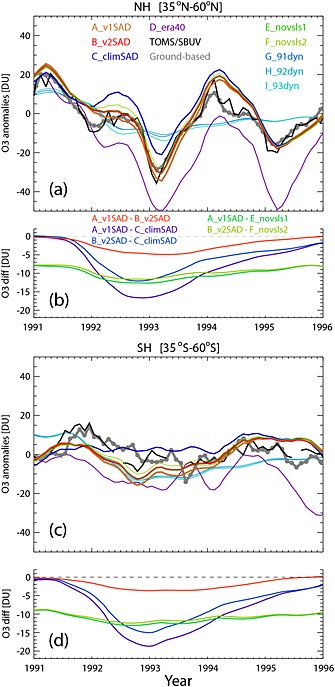
Total O_3_ monthly mean anomalies (DU) from observations and model runs for (a) NH and (c) SH midlatitudes. The anomalies were obtained by subtracting monthly means for 1990–1999. The TOMS/SBUV merged data and ground‐based station data are shown with the solid black and grey lines, respectively. The thick colored lines show anomalies from nine model simulations (see text). The line from run D_era40 has been shifted vertically so that the anomalies match run A_v1SAD in 1991. (b, d) The difference in column O_3_ (DU) between five pairs of model runs in the same latitude regions.

The impact of Mount Pinatubo is often analyzed through monthly mean total O_3_ anomalies. Figure 3 shows this quantity from our model simulations; TOMS/SBUV merged data and ground‐based observations. Note that for much of 1992 in the SH, anomalies from ground‐based measurements are over 5 DU larger than those from TOMS/SBUV data. The exact cause of this difference is not clear, but one possible explanation is that both ground‐based and satellite measurements have large retrieval errors in the presence of enhanced stratospheric aerosol, and there are large gaps in TOMS data during this period. Both ground‐based and satellite data show the well‐known larger O_3_ losses in the NH midlatitudes compared to the SH.

Interestingly, in the SH midlatitudes both observational data sets show a ~5–8 DU increase immediately after the eruption from July 1991 until December 1991. This increase is not reproduced by the model, although the simulations do capture the rate of column O_3_ change from winter 1991 to winter 1992. These differences might be linked to aerosol‐induced changes in the BD circulation [*Young et al.*, 1994; *Aquila et al.,* 2013] that are not represented in the ECMWF reanalysis data, or a strong El Niño–Southern Oscillation event in 1991. Overall, in the SH midlatitudes modeled column O_3_ anomalies from A_v1SAD and B_v2SAD generally follow the observations. As in Figures 1 and 2, comparison with anomalies from C_climSAD suggests that Pinatubo‐induced aerosol contributed to chemical O_3_ losses until late 1994.

In the NH midlatitudes, TOMS/SBUV data show maximum column O_3_ depletions of up to 10 and 40 DU in December 1991 and January 1993, respectively. O_3_ anomalies from ground‐based measurements are smaller than those from TOMS/SBUV in these two winters. Again, this could be related to errors in the O_3_ column retrieval algorithms in the presence of enhanced aerosol loading. During late 1991, observations show ~8 DU O_3_ depletion in the NH, in contrast to the positive anomalies observed in the SH. These negative anomalies in late 1991 are not captured in any model simulation, suggesting stronger stratospheric transport in ERA‐Interim during this period or that some of the enhanced aerosol loading is not well represented in either SAD data set. As for NO_2_ (Figure 1), NH total O_3_ anomalies seem to be shifted by one month. This could be due to large dynamical variability in the NH and that monthly mean SAD are not sufficient to represent this variability.

We can use the model simulations to separate the dynamical and chemical contributions to O_3_ changes. *Poberaj et al.* [2011] argued that significant SH wave forcing during winters 1991 and 1992 increased O_3_ transport from the tropics to middle to high latitudes. However, our simulation with climatological aerosol loading (C_climSAD) shows a slight decrease in O_3_ during austral winter 1991, indicating that during this period the BD circulation is weaker in ERA‐Interim. On the other hand, in the NH midlatitudes run C_climSAD agrees well with the observations in early 1991. None of the model simulations is able to capture the O_3_ changes in late 1991 in either hemisphere, suggesting that either aerosol or strong El Niño‐induced changes in stratospheric circulation are not captured in ERA‐Interim data during this period. Similarly, run C_climSAD shows a slight increase in O_3_ in the SH winter 1992, but a decrease of up to 20 DU in the NH during winter 1992/1993, suggesting a decrease in planetary wave forcing (or dynamical changes) during this period of enhanced O_3_ depletion. This is in agreement with earlier studies [*Hadjinicolaou et al.*, 1997; *Chipperfield,* 2006].

Anomalies from runs G_91dyn, H_92dyn, and I_93dyn, with annually repeating dynamics, quantify the mean chemical contribution to O_3_ changes. In the SH midlatitudes, the model shows losses of up to 15 and 10 DU in November 1992 and 1993, respectively. However, in the NH the model shows losses of up to 10 DU during March in both 1992 and 1993 which are consistent with those reported in *Telford et al.* [2009].

Studies have shown that inclusion of brominated VSLS enhances O_3_ loss in the presence of volcanically enhanced stratospheric aerosols. Comparing runs A_v1SAD (B_v2SAD**)** and E_novsls1 **(**F_novsls2**)** quantifies the VSLS contribution to O_3_ losses in our model (Figure 3). The presence of VSLS decreases column O_3_ by ~8–13 DU, with the larger values corresponding to the period of enhanced aerosol loading. These VSLS‐related O_3_ losses are smaller than those found in earlier studies [e.g., *Salawitch et al.*, 2005; *Feng et al.*, 2007]. In the case of our model this is due to previous positive biases in lower stratospheric O_3_ (e.g., run D_era40) leading to more destruction in the presence of enhanced stratospheric aerosol loading. Note that the model also shows nearly identical VSLS‐related O_3_ decreases in both hemispheres. This confirms that while VSLS chemistry is important for the overall O_3_ budget, it itself does not explain the interhemispheric asymmetry in O_3_ depletion.

**Figure 4:**
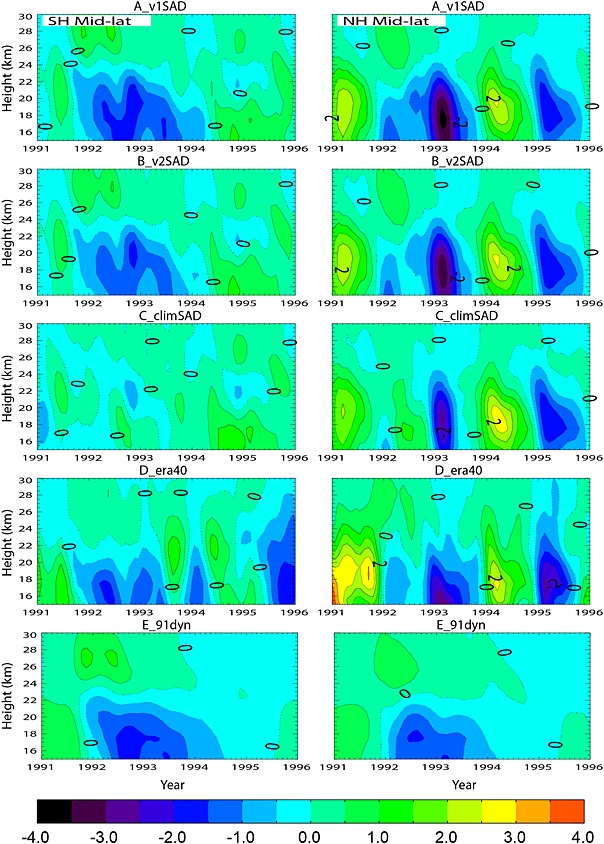
Monthly mean O_3_ anomalies (DU/km) from 1991 to 1995 as a function of altitude from five model simulations for SH midlatitudes (35°S–60°S, left) and NH midlatitudes (35°N–60°N, right). Positive and negative anomalies are represented by solid and dashed lines, respectively. Monthly mean anomalies are calculated by subtracting the climatological 10 year (1990–1999) monthly mean values.

Finally, we analyze changes in the simulated O_3_ profile under different chemical and dynamical conditions. Figure 4 shows monthly anomalies from various model simulations for SH and NH midlatitudes. As total column O_3_ is largely determined by lower stratospheric O_3_, changes in this altitude region are similar to those in Figure 3. In the SH, runs A_v1SAD, B_v2SAD, and C_climSAD all show negative O_3_ anomalies even before the eruption (June 1991) which persist until early 1994. The slight increase in SH total O_3_ anomalies seen in Figure 3 is associated with positive anomalies between 20 and 26 km in mid‐1991. This might be associated with enhanced O_3_ transport from the tropics [*Poberaj et al.*, 2011; *Aquila et al.,* 2013], which is underestimated by ERA‐Interim.

In the NH negative O_3_ anomalies occur from mid‐1991 until late 1993, followed by a sudden increase (~4%) in early 1994. Both runs A_v1SAD and B_v2SAD simulate the largest NH O_3_ depletion (3 DU/km) in the lower stratosphere in early 1993. However, the run with fixed aerosol (C_climSAD) also shows a decrease in O_3_ of nearly 2.5 DU/km in spring 1993, again suggesting a dynamical role in enhancing O_3_ depletion during this period.

A notable feature in Figure 4 from runs A_v1SAD and B_v2SAD is the enhancement in middle stratospheric O_3_ (25–30 km) in both hemispheres immediately after the eruption. This enhancement is also clearly present in run G_91dyn, which shows a larger enhancement in the SH compared to the NH. This effect is absent in C_climSAD and is associated with denoxification (decrease in NO*_x_*) in the presence of stratospheric aerosol causing less chemical O_3_ loss at these altitudes [*Granier and Brasseur*, 1992; *Tie and Brasseur*, 1995]. This enhancement acts to reduce the negative O_3_ column anomaly caused by depletion in the lower stratosphere.

## 5 Summary and Conclusions

We find that use of ERA‐Interim data for dynamical forcing significantly reduces model‐observation biases compared to earlier ERA‐40 reanalyses. Hence, our simulations suggest that estimated Pinatubo‐related dynamical and chemical responses using ERA‐40 data in previous studies [e.g., *Feng et al.*, 2007; *Telford et al.*, 2009] must be carefully interpreted. We also find that simulations with the recently updated SAD [*Arfeuille et al.*, 2013] produce somewhat smaller chemical O_3_ loss during the period of enhanced aerosol than an earlier version of SAD data [*SPARC*, 2006].

The model forced by ERA‐Interim analyses and the updated SAD is able to simulate significant decreases in midlatitude column NO_2_, with the largest changes during December 1991 in the NH and March 1992 in the SH, in good agreement with ground‐based measurements. Simulated O_3_ profiles and total column also show fair agreement with satellite and ground‐based measurements. Our comparison against satellite data also shows smaller background O_3_ in the SH midlatitudes, as has been shown previously [e.g., *WMO*, 2011].

While our updated simulations capture the major aspects of post‐Pinatubo O_3_ depletion, there are some remaining discrepancies. The simulations with ERA‐Interim dynamics are unable to capture the observed increase in SH midlatitude O_3_ immediately after the Pinatubo eruption and the decrease in NH midlatitude O_3_ during winter 1991/1992. This might be related to the lack of aerosol‐induced radiative heating in the ECMWF reanalysis system.

Overall, we conclude that the smaller observed post‐Pinatubo column O_3_ depletion in the SH compared to the NH can be attributed to smaller background O_3_, and enhanced tropics‐to‐high‐latitude transport (via enhanced wave activity and/or through aerosol‐induced heating) during austral winters 1992 and 1993.

## Supporting information



Table S1
