# Supplementary material for: Revisiting the hemispheric asymmetry in midlatitude ozone changes following the Mount Pinatubo eruption: A 3‐D model study
Source: Geophys Res Lett. 2015 Apr 21;42(8):3038–47. doi: 10.1002/2015GL063052 (PMC5102144; doi:10.1002/2015GL063052)
Supplement: Supplementary file 1 — Table S1 [file GRL-42-3038-s001.docx]

Supporting Information for

**Revisiting the hemispheric asymmetry in mid-latitude ozone changes following the Mount Pinatubo eruption: A 3-D model study**

S. S. Dhomse^1^, M. P. Chipperfield^1^, W. Feng^1,2^, R. Hossaini^1^,
G. W. Mann^1,2^ and M. L. Santee^3^

1. School of Earth and Environment, University of Leeds, Leeds, UK.

2. National Centre for Atmospheric Science, University of Leeds, Leeds, U.K.

3. Jet Propulsion Laboratory, California Institute of Technology, Pasadena, California, USA.

**Contents of this file**

**Table S1**

**Introduction**

This supporting information provide summary of dynamical and chemical conditions for model simulations.

**Tables**

| **Experiment** | **Dynamics** | **Aerosol SAD** | **VSLS included?** |
| --- | --- | --- | --- |
| **A_v1SAD** | ERA-int | v1 | Yes |
| **B_v2SAD** | ERA-int | v2 | Yes |
| **C_climSAD** | ERA-int | v1 | Yes |
| **D_era40** | ERA-40 | v1 | Yes |
| **E_novsls1** | ERA-int | v1 | No |
| **F_novsls2** | ERA-int | v2 | No |
| **G_dyn91** | Perpetual Era-int 1991 | v1 | Yes |
| **H_dyn92** | Perpetual Era-int 1992 | v1 | Yes |
| **I_dyn93** | Perpetual Era-int 1993 | v1 | Yes |

**Table S1:** Dynamical and chemical conditions for TOMCAT model simulations.
